# Crystal structure of ethyl 2-cyano-3-[(1-eth­oxy­ethyl­idene)amino]-5-(3-meth­oxy­phen­yl)-7-methyl-5*H*-1,3-thia­zolo[3,2-*a*]pyrimidine-6-carboxyl­ate

**DOI:** 10.1107/S2056989015005241

**Published:** 2015-03-25

**Authors:** M. S. Krishnamurthy, Noor Shahina Begum

**Affiliations:** a Department of Studies in Chemistry, Central College Campus, Bangalore University, Bangalore 560 001, Karnataka, India

**Keywords:** crystal structure, pyrimidine, thia­zolo­pyrimidine, thia­zolo[3,2-*a*]pyrimidine, hydrogen bonding, π–π stacking inter­actions

## Abstract

In the title compound, C_22_H_24_N_4_O_4_S, the central pyrimidine ring adopts a sofa conformation with the ring-junction N atom displaced by 0.2358 (6) Å from the mean plane of the remaining ring atoms. The 3-meth­oxy­phenyl ring, at the chiral C atom opposite the other N atom, is positioned axially and is inclined to the thia­zolo­pyrimidine ring with a dihedral angle of 83.88 (7)°. The thia­zole ring is essentially planar (r.m.s. deviation = 0.0034 Å). In the crystal, pairs of weak C—H⋯O hydrogen bonds link mol­ecules related by twofold rotation axes to form *R*_2_^2^(8) rings, which in turn are linked by weak C—H⋯N inter­actions, forming ribbons along [-110]. In addition, π–π stacking inter­actions [centroid—centroid distance = 3.5744 (15) Å] connect the ribbons, forming slabs lying parallel to (001).

## Related literature   

For background and pharmacological properties of pyrimidine and thia­zolo­pyrimidine derivatives, see: Singh *et al.* (2011 ▸); Ozair *et al.* (2010*a* ▸,*b* ▸); Sayed *et al.* (2010 ▸); Zhi *et al.* (2008 ▸); Mobinikhaledi *et al.* (2005 ▸). For related crystal structures, see: Krishnamurthy & Begum (2014 ▸); Krishnamurthy *et al.* (2014 ▸); Nagarajaiah & Begum (2011 ▸).
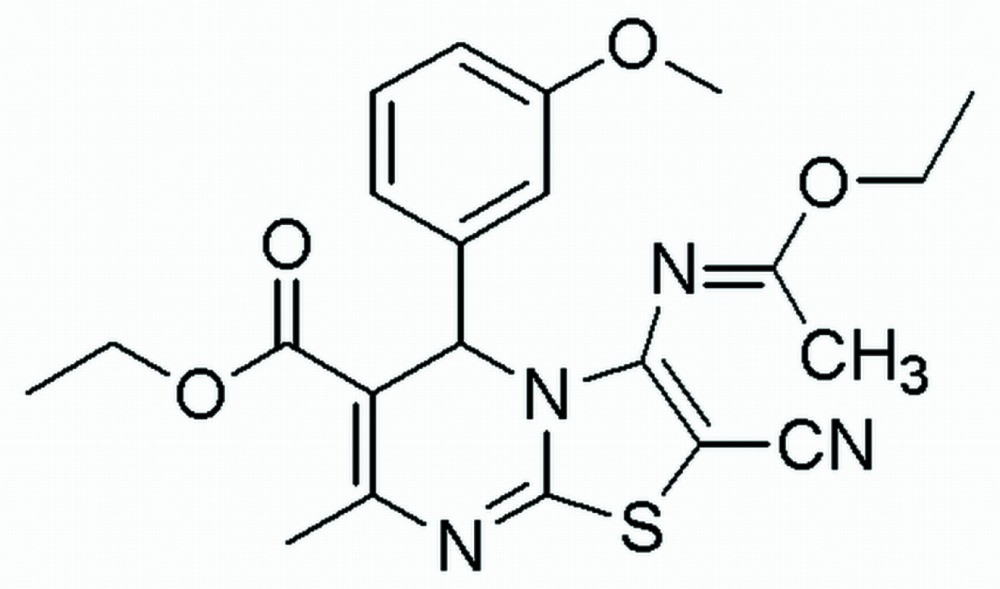


## Experimental   

### Crystal data  

C_22_H_24_N_4_O_4_S
*M* *_r_* = 440.51
Monoclinic, 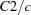

*a* = 14.371 (3) Å
*b* = 13.368 (3) Å
*c* = 22.771 (6) Å
β = 99.325 (5)°
*V* = 4316.9 (16) Å^3^
*Z* = 8
Mo *K*α radiation
μ = 0.19 mm^−1^
*T* = 100 K
0.16 × 0.12 × 0.10 mm

### Data collection  

Bruker SMART APEX CCD detector diffractometer
Absorption correction: multi-scan (*SADABS*; Bruker, 1998 ▸) *T*_min_ = 0.967, *T*_max_ = 0.971
11002 measured reflections
3793 independent reflections
2882 reflections with *I* > 2σ(*I*)
*R*_int_ = 0.052

### Refinement  

*R*[*F* ^2^ > 2σ(*F* ^2^)] = 0.050
*wR*(*F* ^2^) = 0.149
*S* = 1.01
3793 reflections
285 parameters
H-atom parameters constrained
Δρ_max_ = 0.49 e Å^−3^
Δρ_min_ = −0.32 e Å^−3^

### 

Data collection: *SMART* (Bruker, 1998 ▸); cell refinement: *SAINT-Plus* (Bruker, 1998 ▸); data reduction: *SAINT-Plus*; program(s) used to solve structure: *SHELXS97* (Sheldrick, 2008 ▸); program(s) used to refine structure: *SHELXL97* (Sheldrick, 2008 ▸); molecular graphics: *ORTEP-3 for Windows* (Farrugia, 2012 ▸) and *CAMERON* (Watkin *et al.*, 1996 ▸); software used to prepare material for publication: *WinGX* (Farrugia, 2012 ▸).

## Supplementary Material

Crystal structure: contains datablock(s) global, I. DOI: 10.1107/S2056989015005241/su5092sup1.cif

Structure factors: contains datablock(s) I. DOI: 10.1107/S2056989015005241/su5092Isup2.hkl

Supporting information file. DOI: 10.1107/S2056989015005241/su5092Isup3.cml

**Fig. 1.**
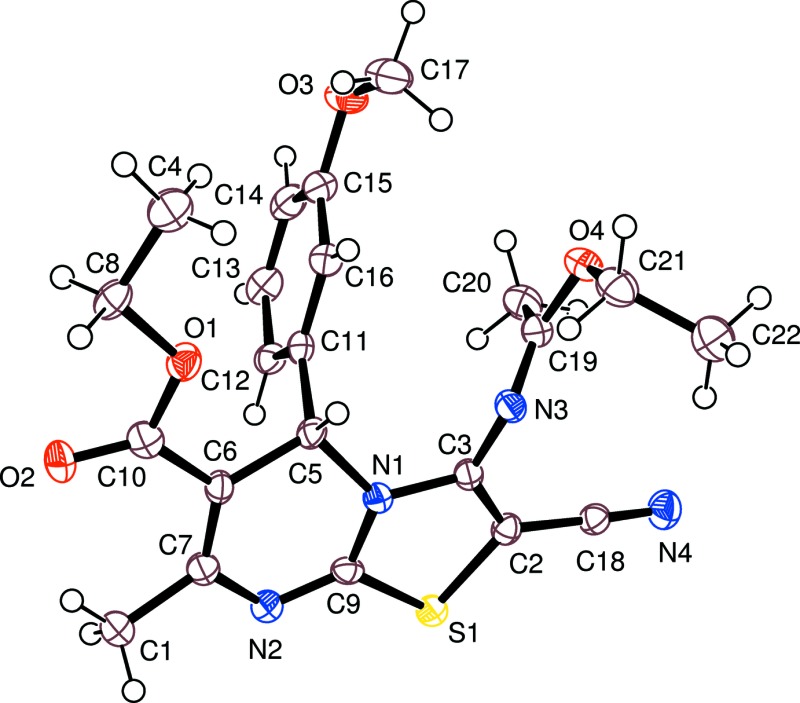
The molecular structure of the title compound, with the atom labelling. Displacement ellipsoids are drawn at the 50% probability level.

. DOI: 10.1107/S2056989015005241/su5092fig1.tif
The mol­ecular structure of the title compound, with the atom labelling. Displacement ellipsoids are drawn at the 50% probability level.

**Fig. 2.**
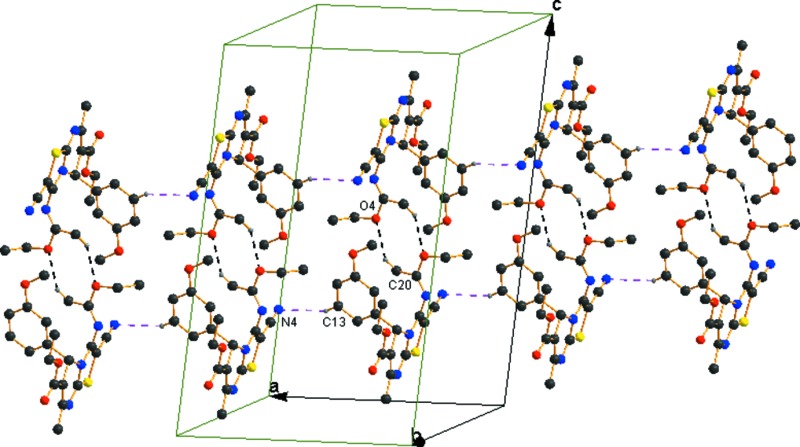
Crystal packing of the title compound viewed along the b axis, showing the intermolecular interactions as dashed lines (see Table 1). H-atoms not involved in hydrogen bonding have been omitted for clarity.

. DOI: 10.1107/S2056989015005241/su5092fig2.tif
Crystal packing of the title compound viewed along the b axis, showing the inter­molecular inter­actions as dashed lines (see Table 1). H-atoms not involved in hydrogen bonding have been omitted for clarity.

CCDC reference: 1054504

Additional supporting information:  crystallographic information; 3D view; checkCIF report

## Figures and Tables

**Table 1:** Hydrogen-bond geometry (, )

*D*H*A*	*D*H	H*A*	*D* *A*	*D*H*A*
C13H13N4^i^	0.95	2.67	3.396(4)	134
C21H21*A*N2^ii^	0.99	2.65	3.538(2)	149
C20H20*B*O4^iii^	0.98	2.68	3.249(5)	117

Symmetry codes: (i) 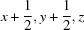; (ii) 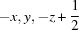; (iii) 
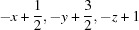
.

## supplementary crystallographic information

### S1. Comment

Pyrimidine has been subjected to a variety of structural modifications in order to synthesize derivatives (Singh *et al.*, 2011) with different biological properties, among which, a thiazole ring fused to a pyrimidine ring, viz. a thiazolopyrimidine, has been found to be more active (Ozair *et al.*, 2010a,b; Sayed *et al.*, 2010). Thiazolo[3,2-*a*]pyrimidine derivatives act as potential enzyme inhibitors and are novel therapeutic entities for severe neurodegenerative diseases (Zhi *et al.*, 2008). In continuation of our research interests on thiazolo[3,2-*a*]pyrimidine derivatives (Krishnamurthy & Begum, 2014; Krishnamurthy *et al.*, 2014), we attempted to synthesize tricyclic thiazolopyrimidine derivatives (Mobinikhaledi *et al.*, 2005). The title compound, an intermediate, was isolated and we report herein on its crystal structure.

The molecular structure of the title compound is shown in Fig. 1. The 3-methoxy phenyl ring at chiral carbon C5 is positioned axially and exactly bisects the thiazolopyrimidine ring with a dihedral angle of 83.88 (7)°. The pyrimidine ring adopts a flattened sofa conformation with atom N1 displaced by 0.2358 (6) Å from the mean plane of the other five atoms (C5/C6/C7/N2/C9). The carbonyl group of the exocyclic ester at C6 adopts a *cis* orientation with respect to C6—C7 double bond. The 3-methoxy phenyl ring adopts a *syn* periplanar conformation with respect to C5—H5 bond of the pyrimidine ring. The thiazole ring is essentially planar (r.m.s. deviation = 0.0034 Å).

In the crystal, pairs of weak C—H···O hydrogen bonds link molecules related by twofold rotation axes to form *R*_2_^2^(8) rings, which are in turn linked by weak C—H···N interactions to form ribbons along [110]; Table 1 and Fig. 2. In addition, π–π stacking interactions with a centroid—centroid distance of 3.5744 (15) Å connect the ribbons to form slabs lying parallel to (001); [Cg1···.Cg1^i^ where Cg1 is the centroid of ring S1/N1/C2/C3/C9; symmetry code: (i) -x, y, -z+1/2].

### S2. Experimental

A mixture of ethyl 3-amino-2-cyano-5-(3-methoxyphenyl)-7-methyl-5*H*- thiazolo[3,2 a] pyrimidine-6-carboxylate (1.85 g, 5 mmol) and triethylorthoacetate (2 ml) was heated under reflux in acetic anhydride for 6 h. Excess triethylorthoacetate and acetic anhydride was removed. The residue was treated with petroleum ether. The solid that separated was filtered, washed and recrystallized from petroleum ether by slow evaporation, yielding light-greenish yellow crystals suitable for X-ray diffraction studies (yield 83%; m.p.: 384 K).

### S3. Refinement

The H atoms were placed at calculated positions in the riding model approximation: C—H = 0.95 - 1.00 Å with *U*_iso_(H) = 1.5*U*_eq_(C) for methyl H atoms and = 1.2*U*_eq_(C) for other H atoms.

### Crystal data

**Table:**

C_22_H_24_N_4_O_4_S	*F*(000) = 1856
*M**_r_* = 440.51	*D*_x_ = 1.356 Mg m^−^^3^
Monoclinic, *C*2/*c*	Mo *K*α radiation, λ = 0.71073 Å
Hall symbol: -C 2yc	Cell parameters from 3793 reflections
*a* = 14.371 (3) Å	θ = 1.8–25.0°
*b* = 13.368 (3) Å	µ = 0.19 mm^−^^1^
*c* = 22.771 (6) Å	*T* = 100 K
β = 99.325 (5)°	Block, yellow
*V* = 4316.9 (16) Å^3^	0.16 × 0.12 × 0.10 mm
*Z* = 8	

### Data collection

**Table:**

Bruker SMART APEX CCD detector diffractometer	3793 independent reflections
Radiation source: fine-focus sealed tube	2882 reflections with *I* > 2σ(*I*)
Graphite monochromator	*R*_int_ = 0.052
ω scans	θ_max_ = 25.0°, θ_min_ = 1.8°
Absorption correction: multi-scan (*SADABS*; Bruker, 1998)	*h* = −17→17
*T*_min_ = 0.967, *T*_max_ = 0.971	*k* = −15→15
11002 measured reflections	*l* = −27→19

### Refinement

**Table:**

Refinement on *F*^2^	Primary atom site location: structure-invariant direct methods
Least-squares matrix: full	Secondary atom site location: difference Fourier map
*R*[*F*^2^ > 2σ(*F*^2^)] = 0.050	Hydrogen site location: inferred from neighbouring sites
*wR*(*F*^2^) = 0.149	H-atom parameters constrained
*S* = 1.01	*w* = 1/[σ^2^(*F*_o_^2^) + (0.1696*P*)^2^] where *P* = (*F*_o_^2^ + 2*F*_c_^2^)/3
3793 reflections	(Δ/σ)_max_ < 0.001
285 parameters	Δρ_max_ = 0.49 e Å^−^^3^
0 restraints	Δρ_min_ = −0.31 e Å^−^^3^

### Special details

**Table:**

Geometry. All s.u.'s (except the s.u. in the dihedral angle between two l.s. planes) are estimated using the full covariance matrix. The cell s.u.'s are taken into account individually in the estimation of s.u.'s in distances, angles and torsion angles; correlations between s.u.'s in cell parameters are only used when they are defined by crystal symmetry. An approximate (isotropic) treatment of cell s.u.'s is used for estimating s.u.'s involving l.s. planes.
Refinement. Refinement of *F*^2^ against ALL reflections. The weighted *R*-factor *wR* and goodness of fit *S* are based on *F*^2^, conventional *R*-factors *R* are based on *F*, with *F* set to zero for negative *F*^2^. The threshold expression of *F*^2^ > 2σ(*F*^2^) is used only for calculating *R*-factors(gt) *etc*. and is not relevant to the choice of reflections for refinement. *R*-factors based on *F*^2^ are statistically about twice as large as those based on *F*, and *R*- factors based on ALL data will be even larger.

### Fractional atomic coordinates and isotropic or equivalent isotropic displacement parameters (Å^2^)

**Table:**

	*x*	*y*	*z*	*U*_iso_*/*U*_eq_	
S1	0.13934 (4)	0.70794 (4)	0.20570 (3)	0.0237 (2)	
O1	0.10595 (11)	1.16153 (12)	0.30234 (7)	0.0258 (4)	
O2	0.12547 (12)	1.21281 (12)	0.21099 (8)	0.0309 (5)	
O3	0.33654 (13)	1.03011 (15)	0.48409 (8)	0.0406 (5)	
O4	0.11827 (12)	0.80604 (12)	0.46167 (7)	0.0277 (4)	
N1	0.11799 (13)	0.86522 (14)	0.26730 (8)	0.0204 (5)	
N2	0.13810 (13)	0.89673 (15)	0.16753 (9)	0.0240 (5)	
N3	0.08826 (13)	0.80714 (14)	0.36078 (9)	0.0219 (5)	
N4	0.12362 (15)	0.52819 (16)	0.33506 (10)	0.0319 (5)	
C1	0.11149 (19)	1.05852 (19)	0.12197 (11)	0.0336 (7)	
H1A	0.1037	1.1294	0.1311	0.050*	
H1B	0.0551	1.0346	0.0957	0.050*	
H1C	0.1667	1.0505	0.1021	0.050*	
C2	0.12358 (16)	0.69605 (17)	0.28022 (11)	0.0216 (5)	
C3	0.11295 (15)	0.78635 (17)	0.30573 (10)	0.0200 (5)	
C4	0.0977 (2)	1.2676 (2)	0.38543 (12)	0.0420 (7)	
H4A	0.0383	1.2355	0.3905	0.063*	
H4B	0.0978	1.3371	0.3992	0.063*	
H4C	0.1505	1.2315	0.4087	0.063*	
C5	0.13384 (16)	0.96962 (16)	0.28873 (10)	0.0197 (5)	
H5	0.0840	0.9878	0.3128	0.024*	
C6	0.12340 (16)	1.03689 (17)	0.23357 (10)	0.0211 (5)	
C7	0.12523 (15)	0.99914 (18)	0.17838 (10)	0.0225 (6)	
C8	0.10788 (18)	1.26578 (17)	0.32076 (11)	0.0275 (6)	
H8A	0.0555	1.3031	0.2968	0.033*	
H8B	0.1682	1.2973	0.3151	0.033*	
C9	0.13237 (15)	0.83739 (18)	0.21150 (10)	0.0208 (5)	
C10	0.11810 (16)	1.14516 (19)	0.24543 (11)	0.0245 (6)	
C11	0.23017 (16)	0.97772 (17)	0.32810 (10)	0.0216 (5)	
C12	0.31098 (16)	0.95397 (16)	0.30541 (11)	0.0238 (6)	
H12	0.3070	0.9350	0.2649	0.029*	
C13	0.39864 (18)	0.95799 (18)	0.34235 (12)	0.0299 (6)	
H13	0.4543	0.9425	0.3267	0.036*	
C14	0.40474 (18)	0.98420 (19)	0.40119 (12)	0.0329 (7)	
H14	0.4644	0.9862	0.4262	0.039*	
C15	0.32356 (19)	1.00781 (19)	0.42400 (11)	0.0301 (6)	
C16	0.23615 (17)	1.00501 (18)	0.38753 (10)	0.0256 (6)	
H16	0.1807	1.0217	0.4031	0.031*	
C17	0.2546 (2)	1.0463 (2)	0.51025 (12)	0.0491 (8)	
H17A	0.2234	1.1080	0.4943	0.074*	
H17B	0.2726	1.0524	0.5535	0.074*	
H17C	0.2114	0.9897	0.5011	0.074*	
C18	0.12295 (16)	0.60302 (18)	0.31003 (11)	0.0227 (6)	
C19	0.14560 (17)	0.79047 (17)	0.40873 (11)	0.0239 (6)	
C20	0.24524 (17)	0.7560 (2)	0.41573 (11)	0.0318 (6)	
H20A	0.2485	0.6850	0.4267	0.048*	
H20B	0.2838	0.7951	0.4470	0.048*	
H20C	0.2690	0.7651	0.3781	0.048*	
C21	0.01889 (18)	0.8272 (2)	0.46214 (12)	0.0316 (6)	
H21A	−0.0065	0.8681	0.4270	0.038*	
H21B	0.0122	0.8659	0.4983	0.038*	
C22	−0.0361 (2)	0.7323 (2)	0.46124 (13)	0.0427 (7)	
H22A	−0.0306	0.6946	0.4250	0.064*	
H22B	−0.1025	0.7479	0.4619	0.064*	
H22C	−0.0112	0.6920	0.4962	0.064*	

### Atomic displacement parameters (Å^2^)

**Table:**

	*U*^11^	*U*^22^	*U*^33^	*U*^12^	*U*^13^	*U*^23^
S1	0.0270 (4)	0.0215 (3)	0.0222 (4)	−0.0008 (2)	0.0033 (3)	−0.0006 (2)
O1	0.0300 (10)	0.0192 (9)	0.0288 (10)	0.0005 (7)	0.0063 (8)	−0.0008 (7)
O2	0.0403 (11)	0.0223 (10)	0.0297 (11)	0.0001 (8)	0.0042 (8)	0.0083 (8)
O3	0.0430 (12)	0.0532 (13)	0.0230 (11)	−0.0142 (10)	−0.0023 (9)	−0.0023 (9)
O4	0.0325 (10)	0.0313 (10)	0.0188 (10)	0.0022 (8)	0.0031 (8)	0.0003 (7)
N1	0.0228 (11)	0.0190 (10)	0.0192 (11)	−0.0003 (8)	0.0026 (8)	0.0009 (8)
N2	0.0269 (11)	0.0222 (11)	0.0228 (12)	−0.0013 (9)	0.0038 (9)	−0.0004 (9)
N3	0.0249 (11)	0.0218 (11)	0.0195 (11)	0.0000 (8)	0.0049 (9)	0.0013 (8)
N4	0.0347 (13)	0.0252 (12)	0.0358 (14)	−0.0007 (10)	0.0057 (10)	0.0041 (10)
C1	0.0477 (17)	0.0295 (15)	0.0225 (14)	−0.0074 (13)	0.0017 (12)	0.0030 (11)
C2	0.0189 (12)	0.0208 (13)	0.0247 (14)	−0.0007 (10)	0.0024 (10)	0.0008 (10)
C3	0.0145 (12)	0.0232 (13)	0.0213 (13)	−0.0011 (10)	0.0006 (10)	0.0044 (10)
C4	0.061 (2)	0.0289 (15)	0.0363 (18)	−0.0040 (14)	0.0081 (15)	−0.0063 (13)
C5	0.0239 (13)	0.0141 (12)	0.0215 (13)	0.0001 (9)	0.0048 (10)	−0.0019 (9)
C6	0.0206 (13)	0.0182 (12)	0.0240 (14)	−0.0013 (9)	0.0020 (10)	0.0040 (10)
C7	0.0181 (12)	0.0257 (13)	0.0234 (14)	−0.0003 (10)	0.0019 (10)	0.0021 (10)
C8	0.0292 (14)	0.0169 (12)	0.0362 (16)	0.0005 (11)	0.0042 (12)	−0.0046 (11)
C9	0.0168 (12)	0.0265 (13)	0.0181 (13)	−0.0013 (10)	0.0003 (10)	−0.0015 (10)
C10	0.0169 (12)	0.0290 (14)	0.0270 (14)	0.0028 (10)	0.0017 (10)	0.0032 (11)
C11	0.0263 (13)	0.0146 (12)	0.0235 (14)	−0.0010 (10)	0.0029 (10)	0.0034 (10)
C12	0.0269 (14)	0.0185 (12)	0.0252 (14)	0.0000 (10)	0.0020 (11)	−0.0014 (10)
C13	0.0239 (14)	0.0252 (14)	0.0398 (17)	0.0018 (11)	0.0028 (12)	−0.0015 (11)
C14	0.0306 (15)	0.0237 (14)	0.0401 (18)	−0.0022 (11)	−0.0070 (13)	0.0011 (12)
C15	0.0365 (16)	0.0254 (14)	0.0262 (15)	−0.0052 (12)	−0.0017 (12)	−0.0003 (11)
C16	0.0268 (14)	0.0257 (14)	0.0235 (14)	−0.0032 (11)	0.0015 (11)	0.0014 (11)
C17	0.057 (2)	0.065 (2)	0.0247 (16)	−0.0221 (17)	0.0049 (14)	−0.0069 (14)
C18	0.0241 (13)	0.0210 (13)	0.0231 (14)	−0.0013 (10)	0.0041 (11)	−0.0016 (11)
C19	0.0281 (13)	0.0211 (13)	0.0220 (14)	−0.0015 (10)	0.0026 (11)	0.0019 (10)
C20	0.0316 (15)	0.0353 (15)	0.0269 (15)	0.0035 (12)	−0.0005 (12)	0.0032 (12)
C21	0.0371 (16)	0.0339 (15)	0.0251 (15)	0.0042 (12)	0.0094 (12)	−0.0019 (11)
C22	0.0474 (18)	0.0475 (18)	0.0355 (17)	−0.0082 (15)	0.0139 (14)	−0.0015 (14)

### Geometric parameters (Å, º)

**Table:**

S1—C9	1.740 (2)	C5—H5	1.0000
S1—C2	1.755 (3)	C6—C7	1.359 (3)
O1—C10	1.353 (3)	C6—C10	1.476 (3)
O1—C8	1.454 (3)	C8—H8A	0.9900
O2—C10	1.213 (3)	C8—H8B	0.9900
O3—C15	1.383 (3)	C11—C12	1.382 (3)
O3—C17	1.419 (3)	C11—C16	1.391 (3)
O4—C19	1.343 (3)	C12—C13	1.398 (3)
O4—C21	1.458 (3)	C12—H12	0.9500
N1—C9	1.371 (3)	C13—C14	1.374 (4)
N1—C3	1.380 (3)	C13—H13	0.9500
N1—C5	1.484 (3)	C14—C15	1.388 (4)
N2—C9	1.291 (3)	C14—H14	0.9500
N2—C7	1.409 (3)	C15—C16	1.390 (3)
N3—C19	1.277 (3)	C16—H16	0.9500
N3—C3	1.385 (3)	C17—H17A	0.9800
N4—C18	1.151 (3)	C17—H17B	0.9800
C1—C7	1.496 (3)	C17—H17C	0.9800
C1—H1A	0.9800	C19—C20	1.488 (3)
C1—H1B	0.9800	C20—H20A	0.9800
C1—H1C	0.9800	C20—H20B	0.9800
C2—C3	1.359 (3)	C20—H20C	0.9800
C2—C18	1.418 (3)	C21—C22	1.492 (4)
C4—C8	1.503 (4)	C21—H21A	0.9900
C4—H4A	0.9800	C21—H21B	0.9900
C4—H4B	0.9800	C22—H22A	0.9800
C4—H4C	0.9800	C22—H22B	0.9800
C5—C11	1.526 (3)	C22—H22C	0.9800
C5—C6	1.532 (3)		
			
C9—S1—C2	89.92 (11)	O2—C10—C6	126.9 (2)
C10—O1—C8	115.58 (18)	O1—C10—C6	110.6 (2)
C15—O3—C17	117.4 (2)	C12—C11—C16	120.0 (2)
C19—O4—C21	117.78 (18)	C12—C11—C5	120.1 (2)
C9—N1—C3	114.3 (2)	C16—C11—C5	119.8 (2)
C9—N1—C5	121.45 (19)	C11—C12—C13	119.7 (2)
C3—N1—C5	122.10 (19)	C11—C12—H12	120.1
C9—N2—C7	115.8 (2)	C13—C12—H12	120.1
C19—N3—C3	121.0 (2)	C14—C13—C12	120.4 (2)
C7—C1—H1A	109.5	C14—C13—H13	119.8
C7—C1—H1B	109.5	C12—C13—H13	119.8
H1A—C1—H1B	109.5	C13—C14—C15	119.9 (2)
C7—C1—H1C	109.5	C13—C14—H14	120.1
H1A—C1—H1C	109.5	C15—C14—H14	120.1
H1B—C1—H1C	109.5	O3—C15—C14	115.6 (2)
C3—C2—C18	124.4 (2)	O3—C15—C16	124.1 (2)
C3—C2—S1	111.95 (18)	C14—C15—C16	120.2 (2)
C18—C2—S1	123.66 (18)	C15—C16—C11	119.7 (2)
C2—C3—N1	112.8 (2)	C15—C16—H16	120.1
C2—C3—N3	128.9 (2)	C11—C16—H16	120.1
N1—C3—N3	117.9 (2)	O3—C17—H17A	109.5
C8—C4—H4A	109.5	O3—C17—H17B	109.5
C8—C4—H4B	109.5	H17A—C17—H17B	109.5
H4A—C4—H4B	109.5	O3—C17—H17C	109.5
C8—C4—H4C	109.5	H17A—C17—H17C	109.5
H4A—C4—H4C	109.5	H17B—C17—H17C	109.5
H4B—C4—H4C	109.5	N4—C18—C2	178.8 (3)
N1—C5—C11	109.64 (18)	N3—C19—O4	119.9 (2)
N1—C5—C6	107.05 (18)	N3—C19—C20	128.5 (2)
C11—C5—C6	113.51 (19)	O4—C19—C20	111.6 (2)
N1—C5—H5	108.8	C19—C20—H20A	109.5
C11—C5—H5	108.8	C19—C20—H20B	109.5
C6—C5—H5	108.8	H20A—C20—H20B	109.5
C7—C6—C10	122.9 (2)	C19—C20—H20C	109.5
C7—C6—C5	121.8 (2)	H20A—C20—H20C	109.5
C10—C6—C5	115.2 (2)	H20B—C20—H20C	109.5
C6—C7—N2	123.1 (2)	O4—C21—C22	110.6 (2)
C6—C7—C1	125.3 (2)	O4—C21—H21A	109.5
N2—C7—C1	111.6 (2)	C22—C21—H21A	109.5
O1—C8—C4	107.32 (19)	O4—C21—H21B	109.5
O1—C8—H8A	110.2	C22—C21—H21B	109.5
C4—C8—H8A	110.3	H21A—C21—H21B	108.1
O1—C8—H8B	110.3	C21—C22—H22A	109.5
C4—C8—H8B	110.2	C21—C22—H22B	109.5
H8A—C8—H8B	108.5	H22A—C22—H22B	109.5
N2—C9—N1	126.2 (2)	C21—C22—H22C	109.5
N2—C9—S1	122.74 (18)	H22A—C22—H22C	109.5
N1—C9—S1	111.07 (17)	H22B—C22—H22C	109.5
O2—C10—O1	122.5 (2)		
			
C9—S1—C2—C3	0.67 (18)	C5—N1—C9—S1	164.04 (16)
C9—S1—C2—C18	−178.8 (2)	C2—S1—C9—N2	−179.1 (2)
C18—C2—C3—N1	178.9 (2)	C2—S1—C9—N1	−0.59 (17)
S1—C2—C3—N1	−0.6 (3)	C8—O1—C10—O2	−3.5 (3)
C18—C2—C3—N3	−8.9 (4)	C8—O1—C10—C6	174.89 (19)
S1—C2—C3—N3	171.65 (19)	C7—C6—C10—O2	−9.0 (4)
C9—N1—C3—C2	0.1 (3)	C5—C6—C10—O2	166.8 (2)
C5—N1—C3—C2	−163.4 (2)	C7—C6—C10—O1	172.7 (2)
C9—N1—C3—N3	−173.04 (18)	C5—C6—C10—O1	−11.5 (3)
C5—N1—C3—N3	23.4 (3)	N1—C5—C11—C12	59.5 (3)
C19—N3—C3—C2	71.3 (3)	C6—C5—C11—C12	−60.2 (3)
C19—N3—C3—N1	−116.8 (2)	N1—C5—C11—C16	−117.8 (2)
C9—N1—C5—C11	−99.3 (2)	C6—C5—C11—C16	122.5 (2)
C3—N1—C5—C11	63.1 (3)	C16—C11—C12—C13	−0.3 (3)
C9—N1—C5—C6	24.2 (3)	C5—C11—C12—C13	−177.6 (2)
C3—N1—C5—C6	−173.38 (19)	C11—C12—C13—C14	0.8 (4)
N1—C5—C6—C7	−15.6 (3)	C12—C13—C14—C15	−0.6 (4)
C11—C5—C6—C7	105.5 (2)	C17—O3—C15—C14	−174.8 (2)
N1—C5—C6—C10	168.50 (18)	C17—O3—C15—C16	3.1 (4)
C11—C5—C6—C10	−70.4 (3)	C13—C14—C15—O3	178.0 (2)
C10—C6—C7—N2	174.6 (2)	C13—C14—C15—C16	0.0 (4)
C5—C6—C7—N2	−1.0 (3)	O3—C15—C16—C11	−177.3 (2)
C10—C6—C7—C1	−7.2 (4)	C14—C15—C16—C11	0.5 (4)
C5—C6—C7—C1	177.2 (2)	C12—C11—C16—C15	−0.3 (3)
C9—N2—C7—C6	11.2 (3)	C5—C11—C16—C15	177.0 (2)
C9—N2—C7—C1	−167.2 (2)	C3—C2—C18—N4	−50 (14)
C10—O1—C8—C4	−177.4 (2)	S1—C2—C18—N4	130 (13)
C7—N2—C9—N1	−2.1 (3)	C3—N3—C19—O4	−176.35 (19)
C7—N2—C9—S1	176.16 (16)	C3—N3—C19—C20	5.0 (4)
C3—N1—C9—N2	178.8 (2)	C21—O4—C19—N3	9.1 (3)
C5—N1—C9—N2	−17.5 (3)	C21—O4—C19—C20	−172.0 (2)
C3—N1—C9—S1	0.4 (2)	C19—O4—C21—C22	84.7 (3)

### Hydrogen-bond geometry (Å, º)

**Table:**

*D*—H···*A*	*D*—H	H···*A*	*D*···*A*	*D*—H···*A*
C13—H13···N4^i^	0.95	2.67	3.396 (4)	134
C21—H21*A*···N2^ii^	0.99	2.65	3.538 (2)	149
C20—H20*B*···O4^iii^	0.98	2.68	3.249 (5)	117

Symmetry codes: (i) *x*+1/2, *y*+1/2, *z*; (ii) −*x*, *y*, −*z*+1/2; (iii) −*x*+1/2, −*y*+3/2, −*z*+1.
