# Supplementary figures and images for: Crystal structure of ethyl 2-cyano-3-[(1-eth­oxy­ethyl­idene)amino]-5-(3-meth­oxy­phen­yl)-7-methyl-5H-1,3-thia­zolo[3,2-a]pyrimidine-6-carboxyl­ate
Source: Acta Crystallogr E Crystallogr Commun. 2015 Mar 25;71(Pt 4):o256–7. doi: 10.1107/S2056989015005241 (PMC4438835; doi:10.1107/S2056989015005241)

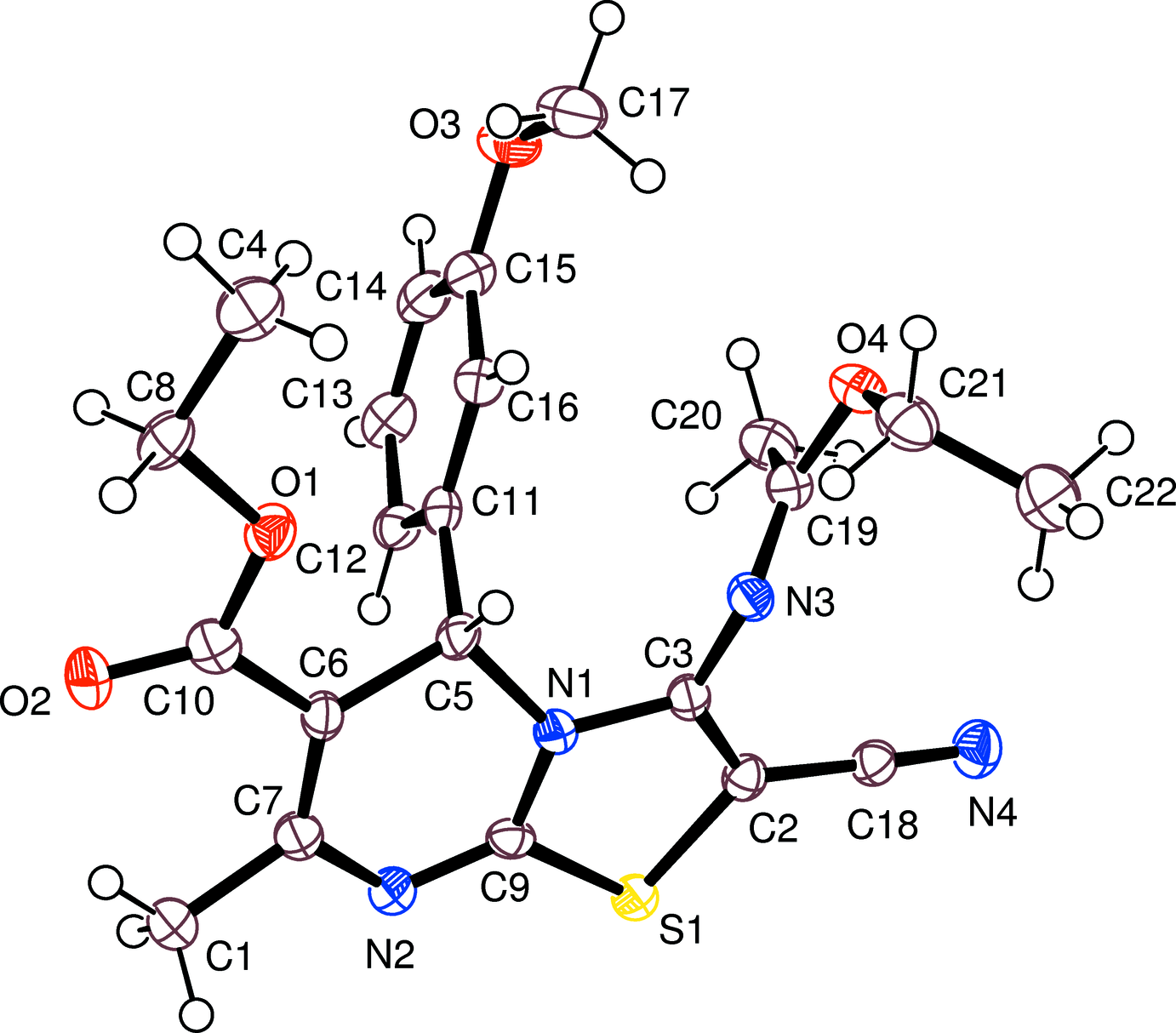

Supplement: Supplementary file 4 [file e-71-0o256-fig1.tif]

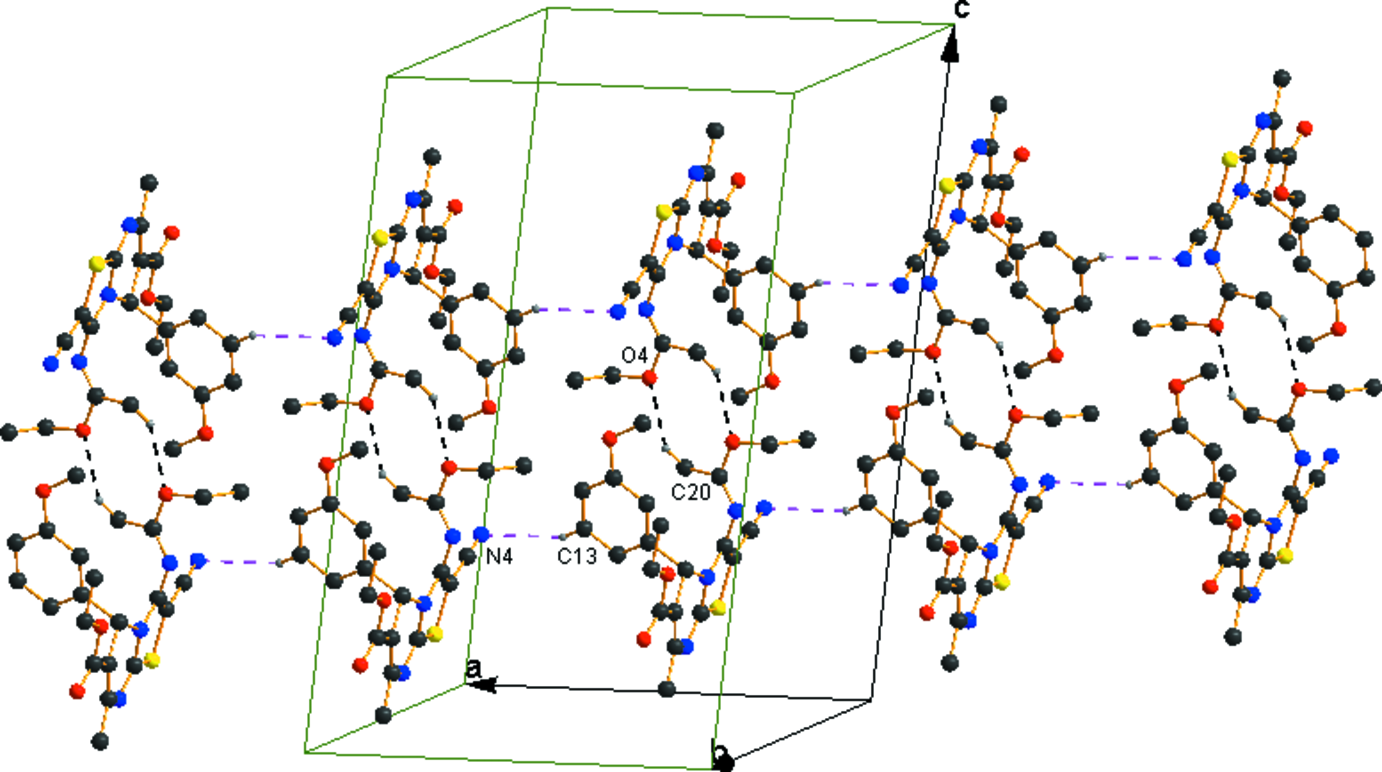

Supplement: Supplementary file 5 [file e-71-0o256-fig2.tif]
